# Weather data analysis and building performance assessment during extreme climate events: A Canadian AMY weather file data set

**DOI:** 10.1016/j.dib.2024.110036

**Published:** 2024-01-09

**Authors:** Milad Rostami, Santinah Green-Mignacca, Scott Bucking

**Affiliations:** Department of Civil and Environmental Engineering, Carleton University, 1125 Colonel By Drive, Ottawa, Ontario, K1S 5B6, Canada

**Keywords:** Climate change, Grid outages, Building performance simulation, AMY weather files

## Abstract

The increasing intensity and frequency of extreme weather events resulting from climate change have led to grid outages and other negative consequences. To ensure the resilience of buildings which serve as primary shelters for occupants, resilient strategies are being developed to improve their ability to withstand these extreme events (e.g., building upgrades and renewable energy generators and storage). However, a crucial step towards creating a resilient built environment is accurately estimating building performance during such conditions using historical extreme climate change-induced weather events. To conduct Building Performance Simulation (BPS) in extreme conditions, such as weather events induced by climate change, it is essential to utilize Actual Meteorological Year (AMY) weather files instead of Typical Meteorological Year (TMY) files. AMY files capture the precise climatic conditions during extreme weather events, enabling accurate simulation of such scenarios. These weather files provide valuable data that can be used to assess the vulnerabilities and resilience of buildings to extreme weather events. By analyzing past events and their impacts using BPS tools, we can gain insights into the specific weaknesses and areas that require improvement. This approach applies to both existing buildings needing climate change-resilient retrofits and new building designs that must be compatible with future climatic conditions. Moreover, the intensification and frequency increase of these extreme weather events makes developing adaptation and resilient-building measures imperative. This involves understanding the potential losses that households may experience due to the intensification of extreme events and developing farsighted coping strategies and climate-proof resilient-building initiatives. However, addressing the knowledge gap caused by the absence of an AMY weather file dataset of extreme events is essential. This will allow for accurate BPS during past extreme climate change-induced weather events. To fill this gap, this article introduces a comprehensive .epw format weather file dataset focusing on historical extreme weather events in Canada. This collection encompasses a diverse array of past extreme climate change occurrences in various locations, with potential for future expansion to include additional locations and countries. This dataset enables energy simulations for different types of buildings and considers a diverse range of historical weather conditions, allowing for better estimation of thermal performance.

Specifications Table

**Table:** 

Subject	Civil and structural engineering
Specific subject area	Actual meteorological year weather data of extreme climate change-induced extreme weather events EnergyPlus Energy Management System (EMS) algorithms for developing power outage situations in energy simulation.
Data format	Filtered data in both .csv and .epw
Type of data	.csv (hourly weather data) .epw (hourly weather data) .json (EMS code for outage event) .ddy (hourly weather data)
Data collection	Raw weather data is collected from Environment and Natural Resources Canada (URL: https://climate.weather.gc.ca/historical_data/search_historic_data_e.html) and is later modified and completed using Element software. The weather data is related to the historical extreme climate change-induced weather events.
Data source location	All collected raw data is extracted from Environmental and Natural Resources Canada.
Data accessibility	Repository name: Extreme weather events AMY weather file Data identification number: doi: 10.17632/pcmnjy69gv.1 Direct URL to data: https://data.mendeley.com/datasets/pcmnjy69gv/1
Related research article	One of the extreme climate change events is used in Bucking et al. (2022), “On modelling of resiliency events using building performance simulation: a multi-objective approach” which proposed a multi-objective approach using thermal resilience, annual net-energy, and life-cycle cost to quantify building performance during grid-outages [1].

## 1 Value of the Data

• This data is crucial for simulating the impact of extreme weather events caused by climate change on building performance. The simulation includes historically recorded weather events in Canada to provide accurate information.
• To quantify short-term resilience, it is essential to have accurate data that reflects the extreme climate change events. AMY weather files provide hourly data on temperature, solar irradiation, humidity, and wind direction and wind speed, which can be used to stress test a building in a historically real condition (e.g., during an extreme weather event).
• This dataset provides the opportunity to evaluate the performance of buildings using standard BPS tools, taking into account diverse extreme climate change occurrences (including both hot and cold events) and considering specific conditions (e.g., power outages).
• Using raw historical weather data in BPS tools is not straightforward, as it requires additional processing to represent extreme climate events accurately (raw materials should be converted into .epw weather files for each event). This dataset enables researchers to easily simulate building performance during such events and allows for accurate energy simulations across various building types and conditions. However, there is limited access to global high-resolution climate data, which poses a challenge in accurately simulating extreme weather events on a regional or local scale.

## 2 Background

To accurately simulate and evaluate building performance during extreme weather events, it is crucial to have access to reliable and customized weather data sets [2]. However, most BPS studies use TMY weather files, which provide average annual weather conditions [3]. This approach fails to capture the impact of extreme climate events on building performance, as these events are often underrepresented or averaged out in typical weather files [4]. Accurate weather data becomes even more essential when assessing building resilience and designing building systems to withstand extreme weather conditions [5]. Therefore, to accurately assess building performance and design resilient systems, it is necessary to use weather data sets that include extreme climate events.

Recent research indicates that extreme cold events persist and affect atmospheric patterns in the Northern Hemisphere, alongside the more prevalent occurrence of heat waves. In particular, ice storms are predicted to rise within colder regions [6]. Furthermore, multiple studies support the increased risk of overheating and discomfort within households due to global warming [7].

## 3 Data Description

**Fig. 1:**
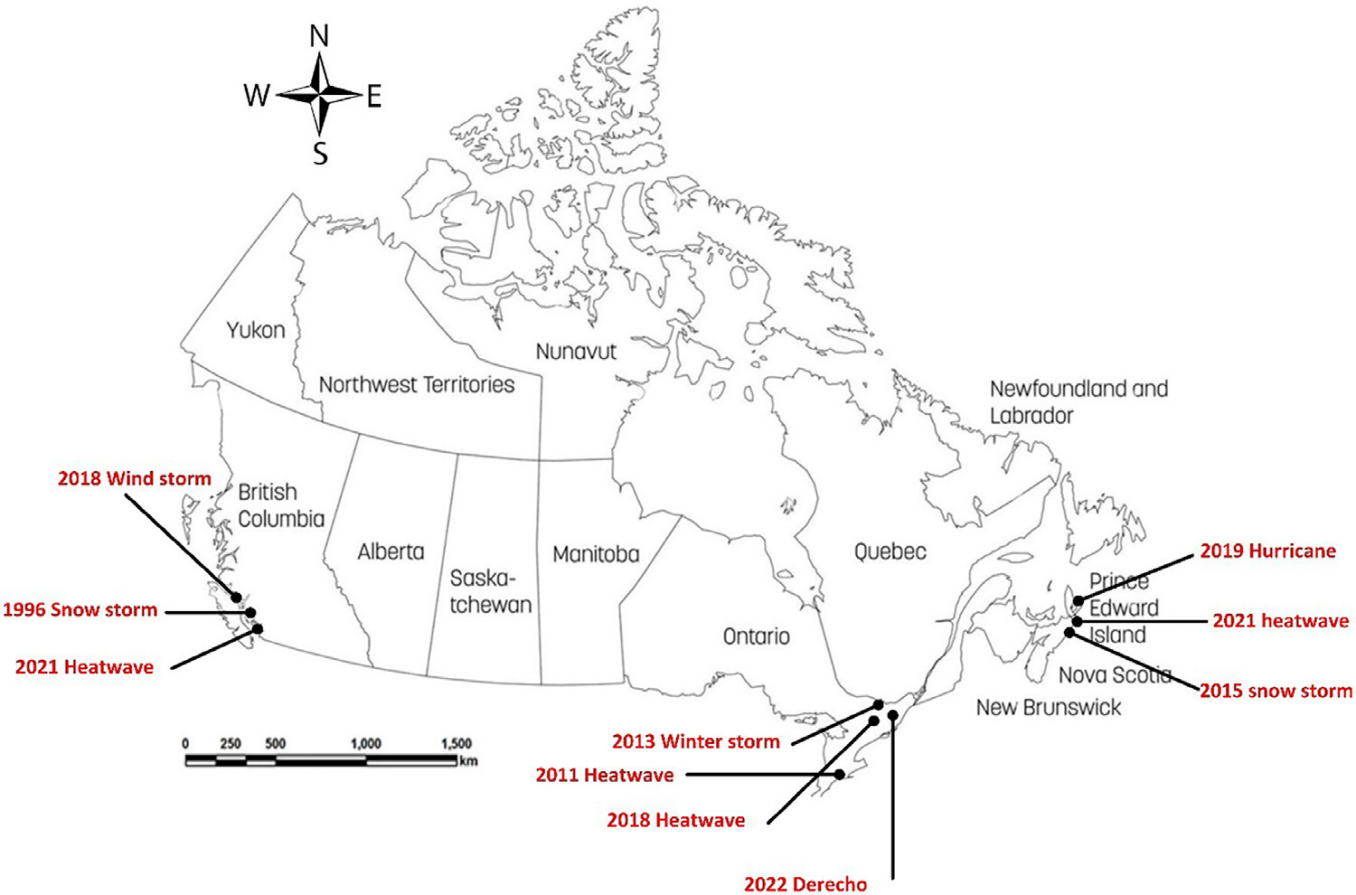
Map of extreme events.

The data set consists of raw historical weather data for Canada, specifically focusing on extreme climate change events. Fig. 1 shows the location and details related to the locations and the extreme weather events. This compilation includes a wide range of previous extreme weather events in different areas, with the potential to expand to more locations and countries.

Fig. 1 illustrates a range of extreme events, such as windstorms, snowstorms, heatwaves, winter storms, hurricanes, and derechos, which are collected to ensure diversity. Extreme weather events have been chosen based on their past frequency and the potential effect they may have on building performance. Another factor in defining extreme events could be their contribution to grid outages, as these events are a leading cause of power failures in North America, with research suggesting that over 80% of these outages can be attributed to such weather occurrences [8] (Table 1).

**Table 1:** Studied extreme weather events.

Weather event	Year	Province	AMY duration	Description
Wind storm	2018/2019	British Columbia (BC)	Dec 5th – Jan 4th	On December 20th, an intense storm resulted in the most significant hydro damage in the province's history. The storm brought over 400 mm of rainfall and wind gusts exceeding 100 km/h [9].
Snowstorm	1996/1997	British Columbia (BC)	Dec 13th – Jan 13th	In December 1996, Victoria experienced a severe blizzard called the “Storm of the Century” by meteorologists at Environment Canada. The city received approximately 124 cm of snow during that month, with an intense snowfall of 65 cm occurring within 24 h on Sunday, Dec. 29th [10]
Heatwave	2021	British Columbia (BC)	June 9th – July 21st	The June 2021 heat wave in British Columbia resulted in the highest fatalities compared to any other disaster in provincial history. This, along with the expenses related to the loss of 619 lives due to heat exposure, makes it one of the most financially burdensome events as well [11]
Winter storm	2013/2014	Ontario (ON)	Dec 7th – Jan 8th	“*The December 2013 North American storm complex was a significant storm complex that included many different types of severe weather, including a winter storm, a crippling ice storm and a tornado outbreak that impacted the central and eastern portions of Canada, parts of the Central Great Plains, the Southern United States, and the northeastern United States*” [12]
Heatwave	2018	Ontario (ON)	June 15th – July 21st	“*From June 29 to July 6, 2018, the air temperature consistently rose above 35* °*C (95.0°F) in parts of Quebec and Ontario. The humidex value for Ottawa on Canada Day between noon and 3 pm was 47.0* °*C (116.6°F), the highest ever recorded in the city*” [13]
Derecho	2022	Ontario (ON)	May 5th – June 6th	The May 2022 Canadian derecho was a significant event that impacted the Quebec City-Windsor Corridor. The event was one of the most influential thunderstorms in Canadian history, with winds reaching up to 190 km/h combined with approximately four tornadoes, resulting in widespread and substantial damage along a path extending 1000 km [14].
Hurricane	2019	Prince Edward Island (PEI)	Aug 21st – Sep 24th	“*Technically, Dorian was done as a hurricane when it landed on P.E.I., but as a post-tropical storm it was still more damaging than 2003′s Hurricane Juan. Dorian dumped about 90 mm s of rain and produced a peak gust of 115* *km/h*” [15].
Heatwave	2021	Prince Edward Island (PEI)	July 27th – Aug 29th	“*There were maximum temperature records set for June 8 in Charlottetown (30.6* °*Celsius (* °*C) breaking the previous record of 27.8* °*C in 1922), Summerside (32.7* °*C breaking the previous record of 27.2* °*C in 1973)*” [16].
Snowstorm	2015	Prince Edward Island (PEI)	Feb 2nd – March 10th	“*Prince Edward Island also set a record in 2015 for the 159* *cm of snow “measured on the ground” at Charlottetown Airport in March, breaking the record from 1956 of 122* *cm. This means that the snow kept accumulating between snowfalls with colder than normal temperatures, rather than melting in between snowfalls*” [16].

As shown in Table 1, “*AMY duration*” demonstrates the time for which the real weather data is included in the .epw file. The duration spans two weeks before and after the event, allowing for precise simulation warm-up procedures before the event and accurate recovery assessments following it.

## 4 Experimental Design, Materials and Methods

AMY weather files should be used to evaluate building performance during historical extreme climate change events. This section describes the development of the AMY weather file dataset to model building performance during past extreme weather events. The AMY dataset for weather files is utilized to model building performance during past extreme weather events with power outages.

Power outages (as an example of a condition that could happen due to an extreme weather event) can be simulated using the capabilities of BPS tools. Algorithm I presents a pseudo-code for the Energy Management System (EMS) in EnergyPlus that enables incorporating outage conditions into simulations (as most BPS tools do not consider power outages). The details about the outage, such as start time, duration, and end time, are specified in a .json file.

**Table:** **Algorithm I**: Outage algorithm in pseudo-code (deployed in Python).

1 # Implementation of override_schedules algorithm to model grid outage with backup
2 function override_schedules(objs,S) #objs is a list of objects, and S is a list of schedules
3 mod_objs = objs #Modifying energy model objects
4 min SoC = 0.1 #Minimum tolerable state of charge in the battery
5
6 mod_objs = add_recov_outage_schedules(mod_objs) #Append grid available and recovery schedules
7
8 i = 0 # Insert template code into EnergyPlus to be called at every timestep (lines 9–21)
9 for s in S do #For each schedule in schedules
10 o = getUserOverrides #Get user-specified schedule overrides
11 mod_objs = add sensor actuator(mod_objs,s) #Append sensor objects
12
13 if SoC ≤minSoC & avail = 0 then #Outage and no battery charge
14 scheoverride = 0
15 else if SoC > min SoC & avail = 0 then #Outage and battery charge
16 scheoverride = o [i][‘batt’]
17 else if avail = 1 & recov = 1 then #Post-event recovery
18 scheoverride = o [i][‘recov’]
19 else pass #Before outage event
20 mod_objs[*i*]['schedule'] = scheoverride #Update mod_objs with the schedule override value
21 i = *i* + 1
22 return mod_objs

### 4.1 Weather files development

**Fig. 2:**
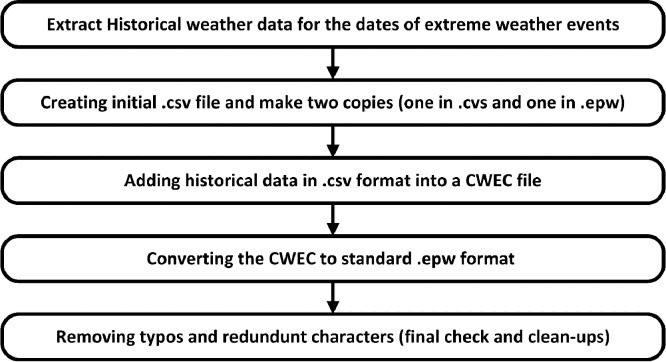
Weather file development.

To begin this process, we gathered raw historical weather data for Canada, focusing on extreme climate change events such as windstorms, snowstorms, heatwaves, winter storms, hurricanes, and derechos, considering various locations to consider multiple climate zones. By including a diverse range of extreme weather conditions, our weather files provide comprehensive coverage for evaluation purposes. To understand each extreme event's distinct features and patterns, we analyzed the collected AMY files (in .cvs format) and cross-referenced them with available reports on these events to validate recorded temperatures. This enabled us to create weather files that accurately depict the duration and intensity of these events. Subsequently, a Canadian Weather-Year for Energy Calculation (CWEC) was utilized to generate standard .epw files for each event while ensuring no typos or other necessary clean-ups or modifications were required. The methodology steps for data development are illustrated in Fig. 2. The following sub-section describes how the weather file could be used to simulate building performance during an extreme climate change-induced weather event, considering power outage as a typical consequence of an extreme event.

### 4.2 The use of weather files for building performance simulation with grid outage

A .json file was developed for the simulation outage to provide specific details about the outage conditions. These details include the start time, duration, and end time of the outage. Additionally, the JSON file contains information about the affected systems and components, such as HVAC, lighting, and electrical systems (Table 2). The .json file is required by the outage algorithm (Algorithm I) that determines the warm-up period, start/stop times, and recovery period.

**Table 2:** JSON file for updating outage time details.

1	“ice-storm”: {
2	“index”: 1,
3	“event_type”: “heating”,
4	“EPW”: “CAN_ON_Ottawa.716280_CWEC_ICESTORM.epw",
5	“t_start”: 854,
6	“t_stop”: 902,
7	“year”: 1998,
8	“Nfreq”: 10,
9	“VoLL_Ccold_exp”: 12.5,
10	“VoLL_Chot_exp”: −0.24375,
11	“sches_turnoff”: {
12	“HVACAvail”: {
13	“sche_name”: “HVAC Avail Sche",
14	“act_batt”: “1.0”
15	“act_recov”: “1.0”
16	},
17	“Light”: {
18	“sche_name”: “APT_LIGHT_SCH",
19	“act_batt”: “0.25”
20	“act_recov”: “1.0”
21	},
22	“Equip”: {
23	“sche_name”: “APT_EQP_SCH",
24	“act_batt”: “0.5”
25	“act_recov”: “1.0”
26	},
27	“DHW”: {
28	“sche_name”: “SWHSys1 Water Heater Setpoint Temperature Schedule Name",
29	“act_recov”: “60”
30	“act_batt”: “0”

This .json file should be updated for each event based on the specific details of that event. For more information regarding the BPS with power outage, please see [1].

To initiate the simulation of building performance during a power outage event, it is necessary to adjust the duration of the simulation run. This adjustment entails reducing the start time by four weeks before the event and extending it until two days post-event. Extending this timeframe may be advisable for larger building case studies to ensure adequate convergence of dynamic models. By default, EnergyPlus is designed to meet specific energy demands irrespective of resource availability. As such, without any modifications (as per version 23.2), EnergyPlus does not possess inherent capabilities for simulating grid outages effectively. To account for temporary power loss in the absence or insufficiency of a battery charge (Algorithm I), adjustments were made to modify the behaviour of the energy modeling tool.

Algorithm I shows the pseudo-code of the outage algorithm. Initially, the outage is modelled by modifying an availability schedule set to “OFF”. However, the fundamental functionality of EnergyPlus is to meet loads and thermostat setpoints through any available sources that caused the necessity to override schedules by directly setting them to zero during the outage (lines 1 to 7). In the case of available charge in the battery system, the load could be met during an outage defined in the pseudo-code in different scenarios (outage & no battery charge, and outage with battery charge (lines 8 to 15)). Finally, the recovery schedule override defines post-event recovery (lines 16 to 18). This method benefits from not relying on building upgrades or specific mechanical system configurations. However, one drawback is that it does not consider other utility outages, such as natural gas. Nevertheless, these could potentially be incorporated using a similar approach. Additionally, the user must provide fractional loads for post-event recovery scheduling.

Moreover, the application of this adapted AMY file extends beyond grid outages and can also be utilized for assessing building performance under various conditions (it is applicable for various operation types and building designs with any size and condition).

## Limitations

One limitation of this dataset is its focus on historical events, which do not account for future climate change projections. However, future climate change-induced extreme events can be projected by adding variations to the weather parameters (e.g., event duration and temperature). Thus, the repository dataset can be expanded to enhance its value in assessing the impact of climate change on building performance during extreme events for future events. Additionally, it is necessary to modify and improve the raw materials using weather file developer tools such as Element before creating the .epw file to accurately include all required weather features, as complete raw data might not be available for all locations.

## Ethics Statement

The authors have read and followed the ethical requirements for publication in Data in Brief and confirmed that the current work does not involve human subjects, animal experiments, or any data collected from social media platforms.

## CRediT authorship contribution statement

**Milad Rostami:** Conceptualization, Methodology, Investigation, Data curation, Writing – original draft. **Santinah Green-Mignacca:** Methodology, Investigation, Resources, Validation. **Scott Bucking:** Project administration, Supervision, Resources, Validation, Software, Conceptualization.

## Data Availability

Extreme weather events AMY weather file (Original data) (Mendeley Data)
